# Investigating the Antibacterial, Antioxidant, and Anti-Inflammatory Properties of a Lycopene Selenium Nano-Formulation: An In Vitro and In Vivo Study

**DOI:** 10.3390/ph17121600

**Published:** 2024-11-27

**Authors:** Reem Binsuwaidan, Thanaa A. El-Masry, Maysa M. F. El-Nagar, Enas I. El Zahaby, Mohamed M. S. Gaballa, Maisra M. El-Bouseary

**Affiliations:** 1 Department of Pharmaceutical Sciences, College of Pharmacy, Princess Nourah bint Abdulrahman University, P.O. Box 84428, Riyadh 11671, Saudi Arabia; rabinsuwaidan@pnu.edu.sa; 2 Department of Pharmacology and Toxicology, Faculty of Pharmacy, Tanta University, Tanta 31527, Egypt; thanaa.elmasri@pharm.tanta.edu.eg; 3 Department of Pharmaceutics, Faculty of Pharmacy, Delta University for Science and Technology, Gamasa 35712, Egypt; enas.elzahabi@deltauniv.edu.eg; 4 Department of Pathology, Faculty of Veterinary Medicine, Benha University, Toukh 13736, Egypt; mohamed.gaballah@fvtm.bu.edu.eg; 5 Department of Microbiology and Immunology, Faculty of Pharmacy, Tanta University, Tanta 31527, Egypt

**Keywords:** lycopene selenium nanoparticles, antibacterial, anti-inflammatory, antioxidant, wound infection

## Abstract

Background: The potent antioxidant lycopene has attracted a large amount of research attention given its potential health benefits. We aimed to assess the antimicrobial, anti-inflammatory, and antioxidant properties of lycopene (Lyc), selenium nanoparticles (Se-NPs), and lycopene selenium nanoparticles (Lyc-Se-NPs). Methods: FTIR, polydispersity index, and zeta potential evaluations provided a complete characterization of the synthesized Lyc-Se-NPs. The broth dilution method and a crystal violet microtiter plate assay were employed to assess the antibacterial and antibiofilm activity, respectively. The rat wound infection model was performed to study the anti-inflammatory effect. Findings: The Lyc-Se-NPs had a zeta potential range of −16.93 to −31.04 mV and a mean particle size of 126.6 ± 3.12 nm. All peaks’ percentage transmittance decreased, according to the FTIR analysis of the Lyc-Se-NPs, with the exception of one peak at 2924.22 cm^−1^, which is suggestive of C-H stretching. The mean scavenging concentrations for Lyc-Se-NPs in the DPPH and ABTS radical scavenging experiments were 3.85 ± 0.65 and 4.26 ± 0.7 µg/mL, respectively. For *S. aureus*, the Lyc-Se-NPs’ MIC values varied from 64 to 1024 µg/mL. CLSM verified that *S. aureus* treated with sub-MICs of Lyc-Se-NPs showed a significant reduction in biofilm formation. Furthermore, the group treated with 50 mg of Lyc-Se-NPs showed the quickest rate of wound healing. They demonstrated a notable elevation of the HO^−1^ content in skin tissues, together with the greatest downregulation of TNF-α, IL-1β, and COX-2. Conclusions: The distinguishing features of Lyc-Se-NPs reveal that this unique compound is a promising antibacterial, antioxidant, and anti-inflammatory agent.

## 1. Introduction

Plants are the greatest source of a wide range of medications, and approximately 80% of people in more economically developed countries use traditional medicine today [1].

Carotenoids are significant phytoconstituents that are thought to be largely responsible for fruits’ and vegetables’ health-protective properties [2].

The carotenoid lycopene is the pigment that provides tomatoes, melons, papayas, and red peppers with their distinctive red hue [3]. Tomatoes are one of the main sources of lycopene [4], which makes up 65–98% of the total weight of carotenoids in tomatoes, depending on the variety [4]. Lycopene is a widely used pigment that is well-liked by the food industry for both its nutritional value and culinary addition status [5]. Moreover, lycopene is a potent antioxidant that has attracted a lot of attention for its related health benefits. For instance, it is well-known for its capacity to neutralize oxidative stress and scavenge free radicals, both of which can be related to the treatment of many chronic illnesses, such as cancer, cardiovascular disease, and age-associated degeneration of eyes [6]. Also, lycopene may have antibacterial properties due to its antioxidant activity. Unfortunately, its use as a treatment option has been highly restricted due to its sensitivity to oxidants, light, and heat; its inability to dissolve in water; its minimal solubility in oils at ambient temperatures; its low bioavailability (10–30% of dietary lycopene) [7]; and the fact that it forms crystals during storage due to its high melting point. However, by using lycopene in the form of polymeric nanoparticles (NPs), these disadvantages of the phytochemical can be overcome. It is commonly recognized that polymeric nanoparticles (NPs) improve a formulation’s solubility, stability, absorption, and drug-loading capability [8].

Selenium is a key element, forming a structural component of antioxidant enzymes including glutathione peroxidase and thioredoxin reducates [9]. As such, it may assume a vital function in preserving health, meaning selenium has garnered increasing attention in recent years [10]. Thyroid hormone metabolism and other critical metabolic processes, as well as the proper operation of the immune system, depend on selenium. The element also prevents oxidative-stress-induced cell death by integrating with antioxidant enzymes. Moreover, selenoproteins (proteins containing selenium) are important for redox control and the elimination of reactive oxygen species (ROS) [11], as well as important to the antioxidant, antimicrobial, and antitumor systems [12].

Traditional selenium compounds are used sparingly since the safe dose level is low, limiting their applicability, but nanometer-sized materials have properties that differ from those of bulk materials, and some of them have found entirely new applications. Elemental selenium is one of these materials [13], with selenium nanoparticles (Se-NPs) found to be a more effective and safe form of selenium that has high bioavailability and low toxicity [14].

Furthermore, a pioneering study demonstrated Se-NPs’ antibacterial activity. The authors found that Se-NPs with a diameter of roughly 100 nm effectively suppress the development of *Staphylococcus aureus* at concentrations as low as 7.8 μg/mL [15]. The majority of their trials also showed significant antibacterial efficiency against *Enterococcus faecalis*, *Streptococcus mutans*, and *Porphyromonas gingivalis*, as well as low toxicity and excellent antioxidant action [15].

Wounds pose a significant worldwide health issue to medical facilities, medical personnel, patients, and their families, with serious social and economic implications [16]. Plants have traditionally been used over the centuries as both preventative and therapeutic measures for the wounded. Many plant concoctions, including the leaves of *Marrubium vulgare* L., *Aloe vera*, and *Elaeis guineensis Jacq*, have been used to treat wounds [17]. When the skin is harmed, the body naturally responds by healing the wound, which involves passing through four predetermined phases—hemostasis, inflammation, proliferation, and remodeling [18]. Since numerous elements are involved, the process of wound healing is complicated. Bacteria and fungi can easily colonize wounds, impeding the healing process. In order to prevent sepsis-causing infections of deeper bodily tissues and blood circulation, physicians advise applying topical antibiotics [19]. However, some topical antibiotics exhibit cytotoxicity, which hinders the healing of wounds. Additionally, wound healing and cleaning may be hampered by antibiotic resistance. As an alternative, given their antibacterial, wound-healing, and biocompatible qualities, medicinal plant formulations can be used with some success [20].

Consequently, this investigation assessed the antibacterial, antioxidant, and anti-inflammatory properties of selenium/lycopene nanoparticles in comparison to the utilization of these two elements independently. To the best of our knowledge, this is the first research to investigate the antibacterial and antibiofilm activity of lycopene and a lycopene-selenium nanoformulation against *S. aureus* clinical isolates.

## 2. Results

### 2.1. Characterization of Lycopene, Selenium Nanoparticles, and Selenium/Lycopene Nanoparticles

In this study, the SeNPs were synthesized by a simple wet chemical approach from ascorbic acid with biocompatibility and good reducing characteristics utilizing poloxamer 407 as a stabilizer.

Dynamic Light Scattering (DLS) analysis revealed a single peak with 94.62 ± 0.79% intensity and a mean particle size of 126.6 ± 3.12 nm. The zeta potential ranged from −16.93 to −31.04 mV, with an average of −23.47 ± 7.11 mV. The average value of the PDI was 0.3271 ± 0.023 (Table 1). The average drug content of the colloidal dispersion was 31.85 ± 3.33 µg/mL.

**Figure 1:**
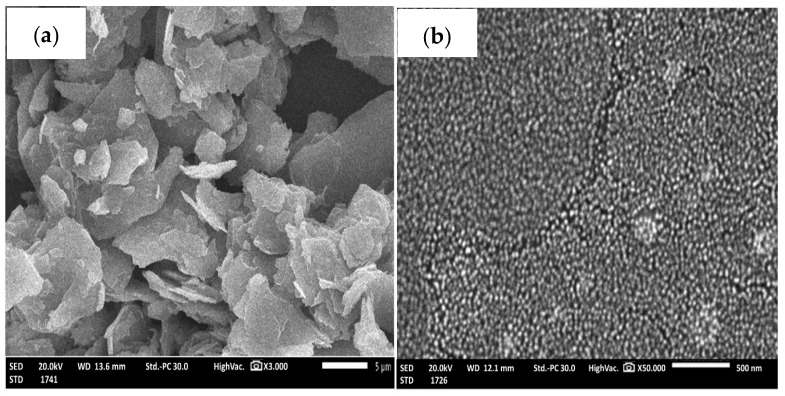

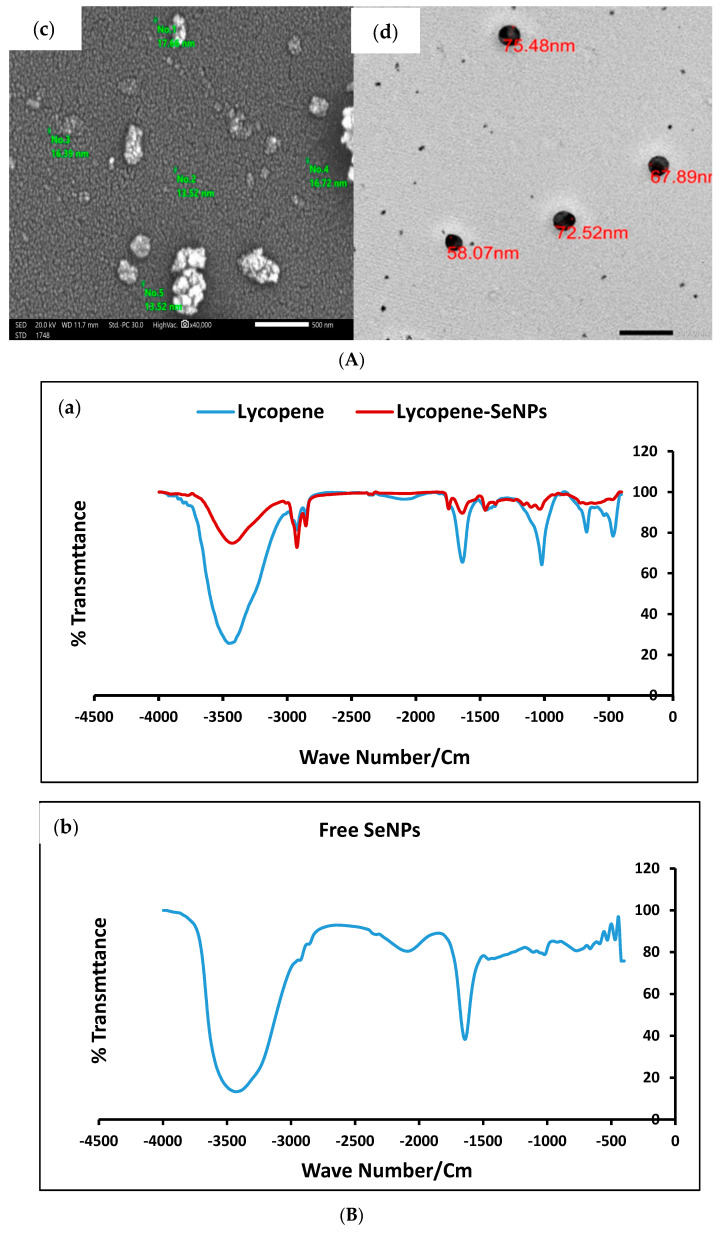
(**A**) SEM of lycopene (**a**), selenium nanoparticles (**b**), and selenium/lycopene nanoparticles (**c**), and TEM of selenium/lycopene nanoparticles (**d**). (**B**) FTIR of lycopene and selenium/lycopene nanoparticles (**a**), and FTIR of Selenium nanoparticles (**b**).

The SEM examination of lycopene (magnification power 3000) illustrated irregular flakes, while selenium nanoparticles (magnification 40,000) contained homogenous spherical particles, and lycopene nanoparticles (magnification 40,000) showed almost the same appearance as selenium nanoparticles (Figure 1A(a,b)). A TEM examination of the lycopene nanoparticles also showed the spherical shape of the nanoparticles, with a particle size less than 100 nm (Figure 1A(d)).

FTIR of lycopene showed the characteristic peak at 2925.21 cm^−1^, which corresponds to C-H stretching. Then, FTIR of selenium/lycopene nanoparticles revealed a decrease in the percentage transmittance of all peaks, except for the peak at 2924.22 cm^−1^, which corresponds to C-H stretching. The FTIR of selenium nanoparticles did not exhibit the C-H stretching at 2925.21 cm^−1^. A characteristic peak was observed at 3285.27 cm^−1^ which represents the hydroxyl group and the peak for C=O stretching around 1700 cm^−1^ (Figure 1B) [21].

### 2.2. Antioxidant Activity Assay

#### 2.2.1. DPPH Radical Scavenging Assay

**Figure 2:**
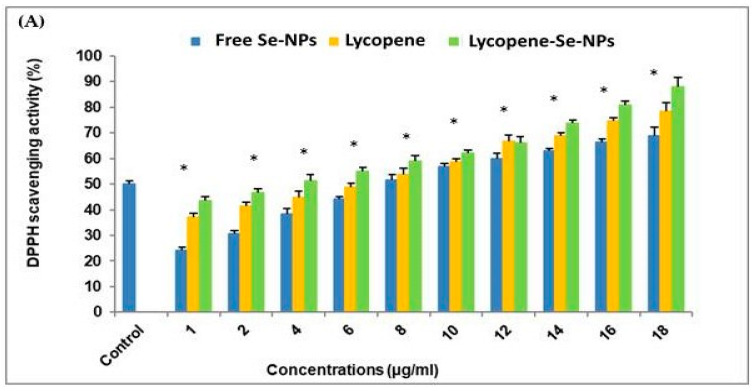

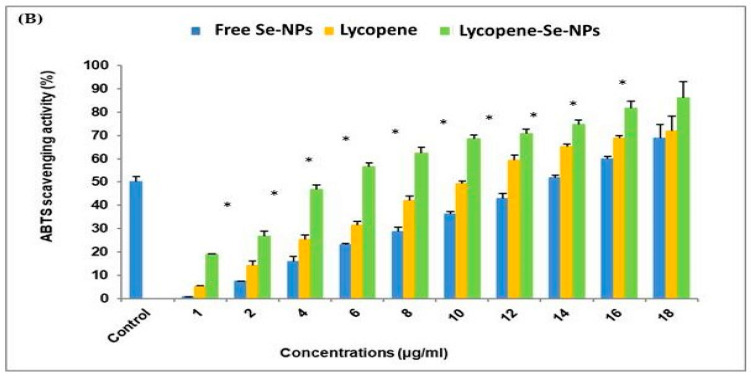
The antioxidant activity of free Se-NPs, lycopene, and Lyc-Se-NPs, evaluated by a DPPH radical scavenging assay (**A**) and ABTS radical scavenging assay (**B**). Results are expressed as the mean ± SD (*n* = 3). Control: ascorbic acid, Se-NPs: selenium nanoparticles. * Significant vs. control group (ascorbic acid) per each concentration (*p* < 0.05).

To assess the antioxidant activity, the DPPH radical scavenging activity was quantified (Figure 2A). Treatments with free Se-NPs, lycopene, and Lyc-Se-NPs were found to generate concentration-dependent DPPH radical scavenging activity (Figure 2A). Lyc-Se-NPs showed superior activity over free Se-NPs and lycopene. The mean scavenging concentrations (IC_50_, expressing 50% of scavenging) were 7.73 ± 1.4, 6.48 ± 0.95, and 3.85 ± 0.65 µg/mL for free Se-NPs, lycopene, and lycopene-Se-NPs, respectively, while the IC_50_ of ascorbic acid as a positive control was 2.53 µg/mL (Figure 2A).

#### 2.2.2. ABTS Radical Scavenging Assay

The DPPH and ABTS assay findings were similar, with the Lyc-Se-NP antioxidant efficacy exceeding that of free Se-NPs and Lyc (Figure 2B). The IC_50_ values of the ABTS scavenging assay were established to be 13.42 ± 1.09, 10.14 ± 2.6, and 4.26 ± 0.7 µg/mL for free Se-NPs, lycopene, and lycopene-Se-NPs, respectively, while the IC_50_ of gallic acid as a positive control was 2.55 µg/mL (Figure 2B).

### 2.3. In Vitro Antibacterial Activity

Lycopene (Lyc) and lycopene selenium nanoparticles (Lyc-Se-NPs) displayed antibacterial activity against the *S. aureus* reference strain, as determined via the agar-well diffusion method (Table 2).

A broth microdilution assay was employed to determine the MIC values of Lyc, selenium, and Lyc-Se-NPs against *S. aureus* isolates (*n* = 20) (see Supplementary Table S1), which ranged from 256 to 1024 µg/mL and from 64 to 1024 µg/mL for Lyc and Lyc-Se-NPs, respectively (Table 3).

### 2.4. Growth Curves of S. aureus Clinical Isolates

**Figure 3:**
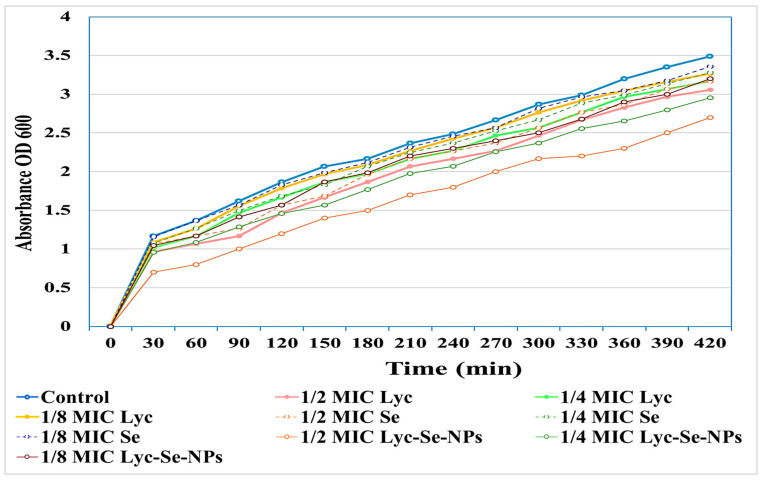
Growth curve of *S. aureus* (S16) clinical isolate cultured in the absence and presence of ½, ¼, and ⅛ MICs of tested compounds at different time intervals.

The impacts of ½, ¼, and ⅛ MICs of the tested compounds on the bacterial growth curve were tested to determine the best concentrations, that is, those that did not affect the growth of bacteria, to be employed in further investigations (see Supplementary Figure S1). Our findings showed that ¼ and ⅛ MICs had no significant effect on the growth of *S. aureus* clinical isolates (Figure 3).

### 2.5. In Vitro Antibiofilm Activity

The total number of biofilm-producing *S. aureus* isolates was reduced from 19 to 11, 16, and 18 following treatments with ½, ¼, and ⅛ MICs of Lyc-Se-NPs, respectively (see Supplementary Table S1). Moreover, these treatments reduced the percentage of strong biofilm-forming isolates from 25% to 5%, 10%, and 15%, respectively (Table 4).

### 2.6. Confocal Laser Scanning Microscopy (CLSM)

**Figure 4:**
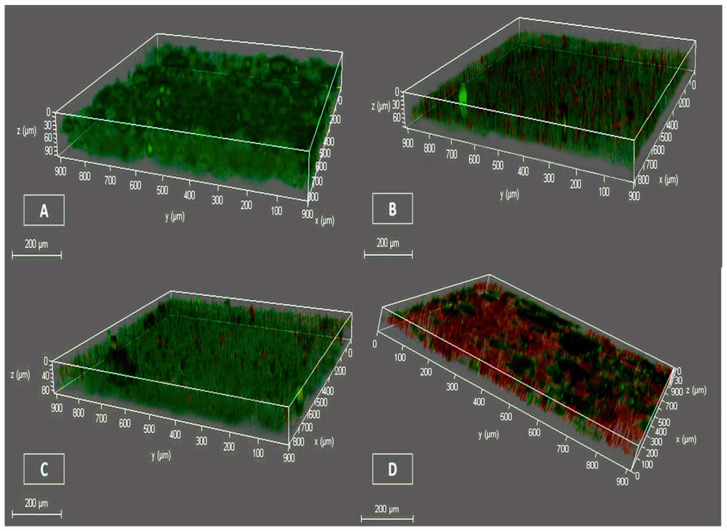
CLSM of *S. aureus* (S16) clinical isolate to detect biofilm thickness pre- and post-treatment with ¼ MIC of the tested compounds. (**A**) Untreated *S. aureus* biofilm. (**B**) Lyc-treated biofilm. (**C**) Selenium-treated biofilm. (**D**) Lyc-Se-NP-treated biofilm.

**Figure 5:**
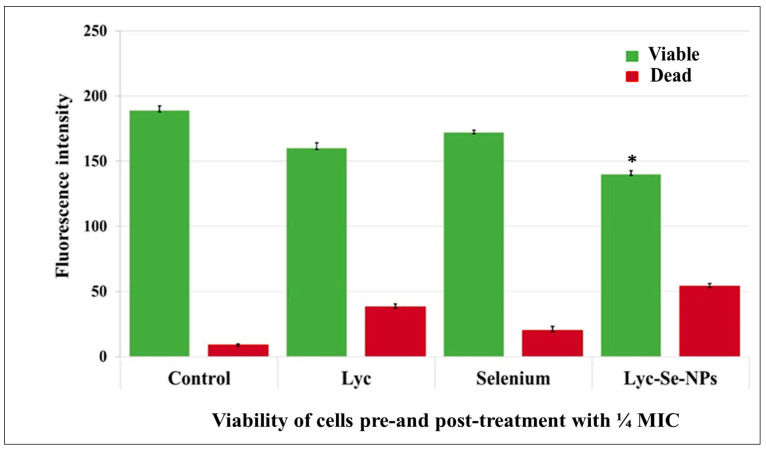
Change in fluorescence intensity with change in viability pre- and post-treatment with ¼ MIC of the tested compounds. * Significant vs. untreated group (*p* < 0.05).

The impact of ¼ MICs of the tested compounds on *S. aureus* biofilm formation was confirmed using CLSM with the double-staining technique (Figure 4). The percentages of reduction after exposure to ¼ MICs of Lyc, selenium, and Lyc-Se-NPs were 33.3, 11, and 66.7%, respectively (Figure 5).

### 2.7. In Vivo Study

#### 2.7.1. Wound Healing

**Figure 6:**
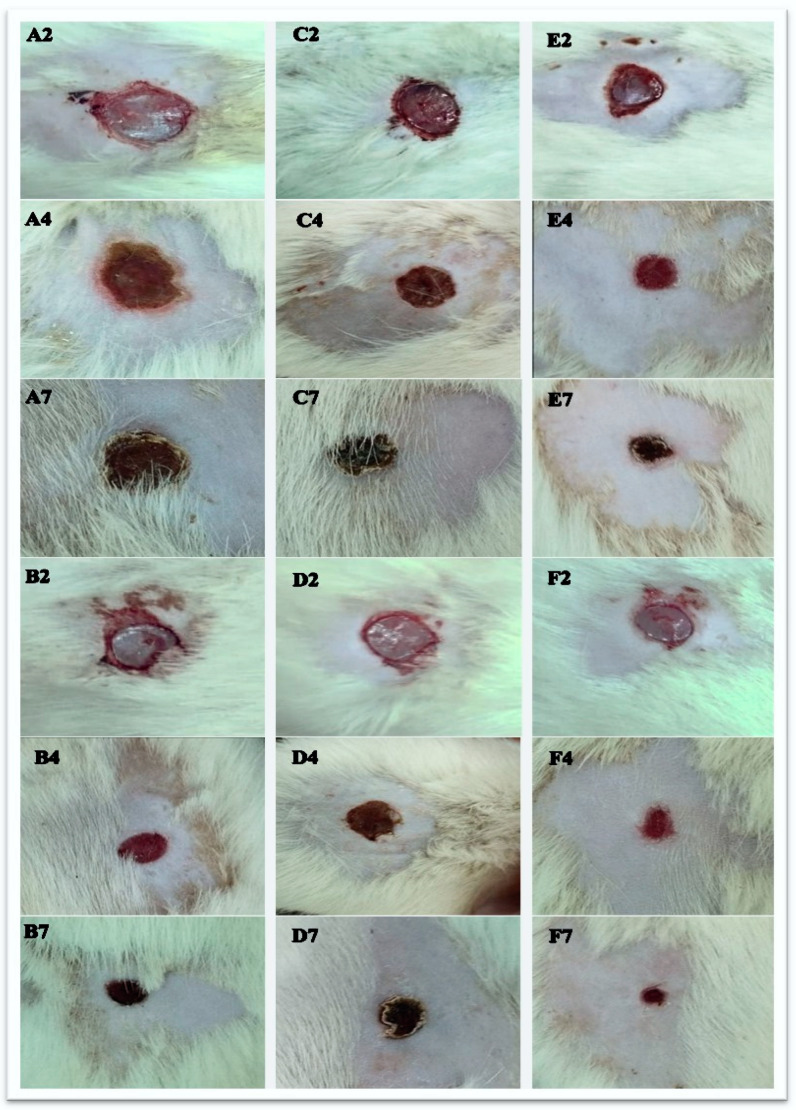
Experimental study of a rat wound-healing model untreated or after treatment with the standard, lycopene only, free Se-NPs, Lyc-Se-NPs (25 mg), or Lyc-Se-NPs (50 mg). The untreated group (**A2**,**A4**,**A7**) exhibited larger wound areas compared to the gentamicin (**B2**,**B4**,**B7**), lycopene-only (**C2**,**C4**,**C7**), free Se-NP (**D2**,**D4**,**D7**), Lyc-Se-NP (25 mg) (**E2**,**E4**,**E7**), and Lyc-Se-NP (50 mg) (**F2**,**F4**,**F7**) groups.

On day two, the wound areas across all groups remained relatively similar, with minimal signs of healing. The untreated (Figure 6(A2)) group exhibited the largest wound areas, showing no notable signs of contraction or healing. In contrast, slight wound contraction was observed in the combination groups, specifically the gentamicin (Figure 6(B2)), Lyc-Se-NP (25 mg) (Figure 6(E2)), and Lyc-Se-NP (50 mg) (Figure 6(F2)) treatment groups. These groups demonstrated early signs of wound healing, albeit marginal, compared to the other groups. The lycopene-only (Figure 6(C2)) and free Se-NP (Figure 6(D2)) groups showed negligible changes in wound size in comparison to the untreated group.

In the same context, on day four, each group showed varying degrees of healing. The wound areas of the gentamicin (Figure 6(B4)), lycopene-only (Figure 6(C4)), free Se-NP (Figure 6(D4)), Lyc-Se-NP (25 mg) (Figure 6(E4)), and Lyc-Se-NP (50 mg) (Figure 6(F4)) treatment groups were notably smaller in comparison to those of the untreated (Figure 6(A4)) group. The Lyc-Se-NP group (50 mg) (Figure 6(F4)) exhibited the most significant reduction in wound size, indicating an accelerated healing process.

As time progressed, on day seven, the wound areas of each group decreased gradually. At this stage, each group displayed varying degrees of wound contraction and scabbing. The lycopene-only (Figure 6(C7)) and free Se-NP (Figure 6(D7)) groups showed significant healing, resulting in smaller wound areas compared to the untreated (Figure 6(A7)) group, but less than the gentamicin (Figure 6(B7)) group. The Lyc-Se-NP (25 mg) (Figure 6(E7)) and Lyc-Se-NP (50 mg) (Figure 6(F7)) groups demonstrated marked reductions in wound size compared to both the untreated (Figure 6(A7)) and gentamicin (Figure 6(B7)) groups, with the Lyc-Se-NP group (50 mg) (Figure 6(F7)) displaying the most advanced healing signs. Over time, all treatment groups showed a reduction in wound areas, with varying degrees of contraction and scabbing. The groups are arranged in order of healing efficacy as follows: Lyc-Se-NPs (50 mg) > Lyc-Se-NPs (25 mg) > gentamicin > lycopene only > free Se-NPs.

The Lyc-Se-NP (50 mg) treated group exhibited the fastest wound healing process, achieving near-complete healing within six days post-wounding in comparison to the control group. The wound regions in the Lyc-Se-NP group (50 mg) were nearly covered with epidermis, and the wound areas were almost completely closed. The average wound closure rate (WCR) is displayed in Table 5.

#### 2.7.2. Impacts of Different Treatments on Inflammatory Markers (TNF-α, IL-1β, and COX-2) Contents in Skin Tissues

**Figure 7:**
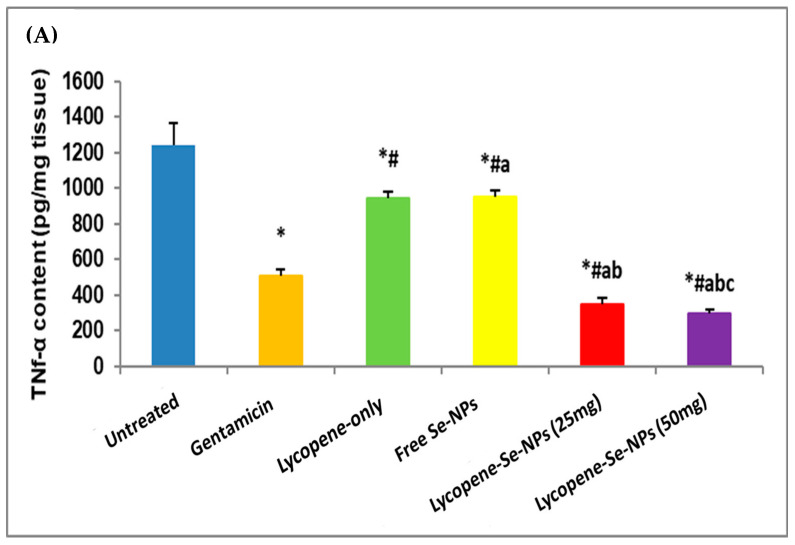

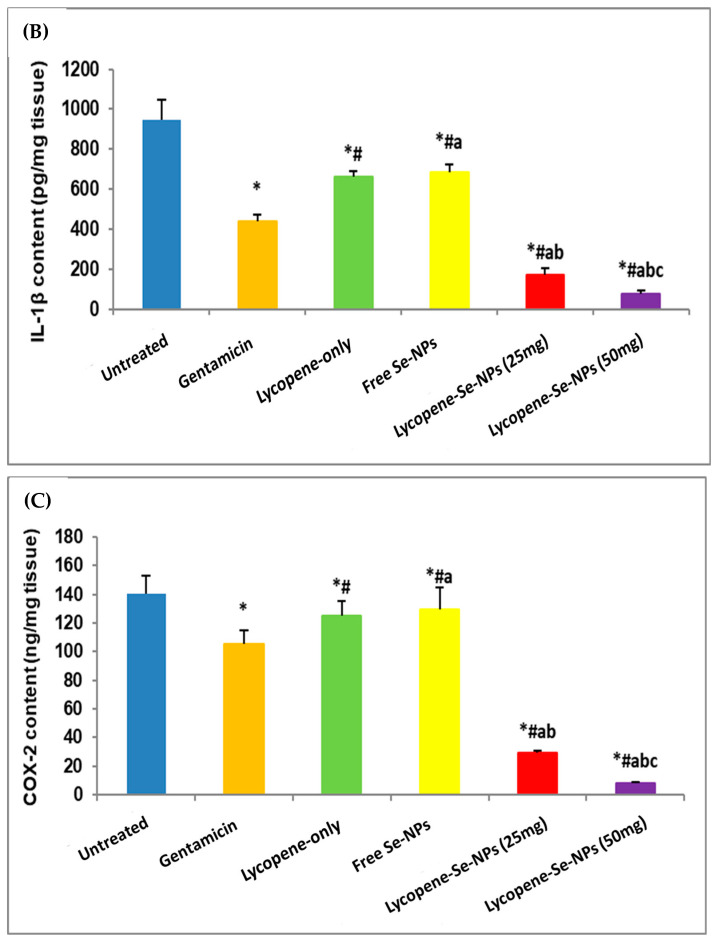
Impacts of different treatments on contents of inflammatory markers TNF-α (**A**), IL-1β (**B**), and COX-2 (**C**) in skin tissues. Data were recorded as mean ± SD (*n* = 6). * Significant vs. untreated group, ^#^ significant vs. gentamicin group, ^a^ significant vs. lycopene-only group, ^b^ significant vs. free Se-NP group, and ^c^ Lyc-Se-NPs (25 mg). Se-NPs: selenium nanoparticles. Each group differed significantly from the others at *p* < 0.05.

The results showed that the contents of inflammatory markers (TNF-α, IL-1β, and COX-2) declined in all treatment groups, but to varying degrees (Figure 7A–C). The Lyc-Se-NP (50 mg) treatment demonstrated the most pronounced downregulating effects (Figure 7A–C).

Lyc-Se-NPs (50 mg) and Lyc-Se-NPs (25 mg) induced a significant reduction in the levels of inflammatory markers TNF-α (76.34 and 71.81%, respectively), IL-1β (892.04 and 82.31%, respectively), and COX-2 (94.35 and 79.42%, respectively) in comparison with the untreated control group. In addition, the gentamicin-treated group displayed notable anti-inflammatory activity, as revealed by a marked decrease in TNF-α, IL-1β, and COX-2 levels (58.84%, 53.75%, and 25%, respectively) compared to the untreated group (Figure 7A–C).

Furthermore, Lyc-Se-NPs (50 mg) and Lyc-Se-NPs (25 mg) brought about a significant reduction in the levels of inflammatory markers TNF-α, IL-1β, and COX-2 compared to the lycopene-only and free Se-NP groups (Figure 7A–C).

#### 2.7.3. Impacts of Different Treatments on the Anti-Inflammatory Marker (HO-1) Content in Skin Tissues

**Figure 8:**
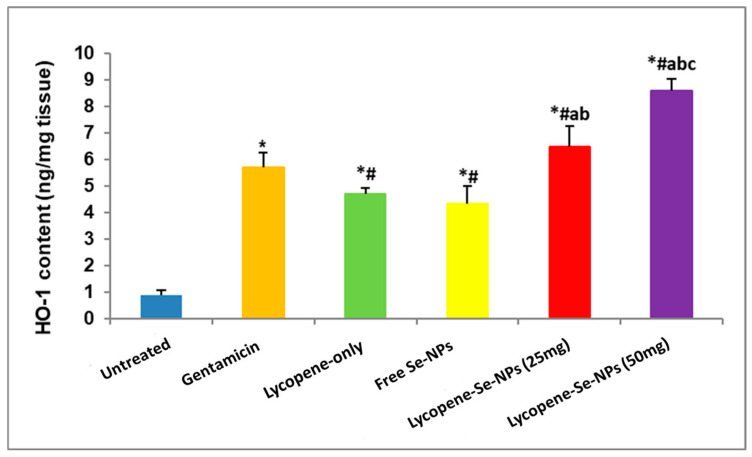
Impacts of different treatments on anti-inflammatory marker (HO-1) content in skin tissues. Data were recorded as mean ± SD (*n* = 6). * Significant vs. untreated group, ^#^ significant vs. gentamicin group, ^a^ significant vs. lycopene-only group, ^b^ significant vs. free Se-NP group, and ^c^ Lyc-Se-NPs (25 mg). Se-NPs: selenium nanoparticles. Each group differed significantly from the others at *p* < 0.05.

According to the results, all treatment groups showed an increase in anti-inflammatory marker (HO-1) content, but to varying degrees (Figure 8). Lyc-Se-NP (50 mg) treatment produced the most notable downregulating effects. Figure 8 shows that treatments with gentamicin, lycopene only, free Se-NPs, Lyc-Se-NPs (25 mg), and Lyc-Se-NPs (50 mg) produced remarkable amplification of 4.67, 3.74, 3.38, 5.54, and 7.67-fold, respectively, in HO-1 content compared to the untreated group (Figure 8). In addition, treatment with Lyc-Se-NPs (50 mg) produced substantial increases of 82.94%, 97.69%, and 32.61% in HO-1 content compared to lycopene only, free Se-NPs, and Lyc-Se-NPs (25 mg), respectively (Figure 8).

#### 2.7.4. Histopathological Evaluation of Skin

**Figure 9:**
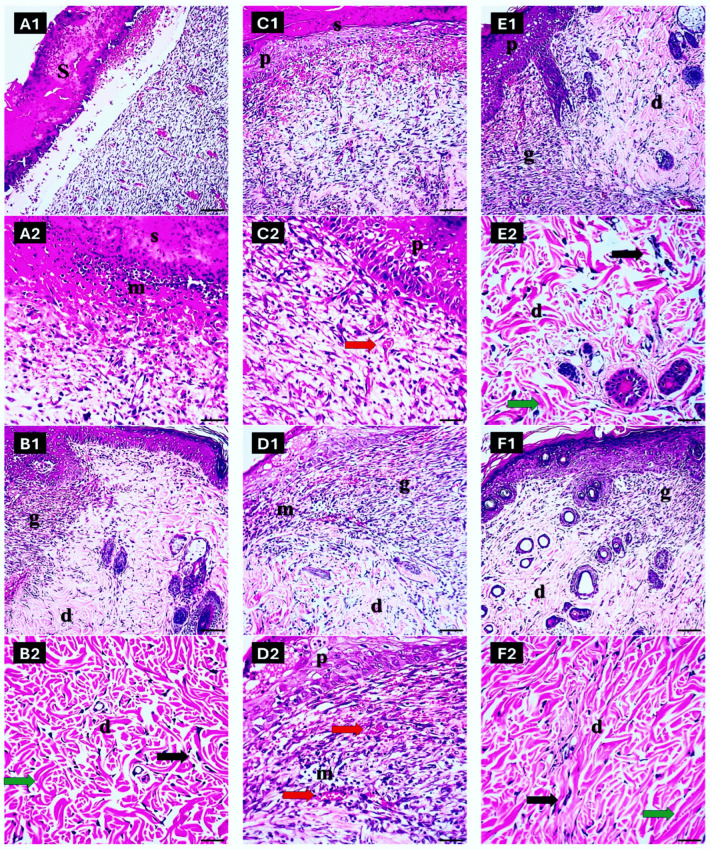
Histopathological examination of wound healing and tissue integrity across different study groups. In the untreated group (**A1**,**A2**), delayed wound healing is evident, characterized by persistent fibrin deposition, absence of granulation tissue, and extensive inflammatory cell infiltration. The epidermis shows necrotic areas and disrupted epithelial integrity, while the dermis exhibits severe inflammatory infiltration with neutrophils and macrophages. The gentamicin group (**B1**,**B2**) demonstrates advanced healing with initial granulation tissue formation and early collagen deposition. The epidermis displays moderate re-epithelialization, and the dermis shows a significantly reduced inflammatory response. In the lycopene-only group (**C1**,**C2**), mild preservation of epidermal architecture and reduced necrosis can be observed. Enhanced granulation tissue formation and improved collagen organization indicate accelerated wound closure. The free Se-NP group (**D1**,**D2**) similarly shows preserved epidermal layers, mild inflammatory infiltrate (predominantly lymphocytic), and increased vascularization and collagen synthesis at the wound site, suggesting improved early tissue repair. The Lyc-Se-NP (25 mg) group (**E1**,**E2**) exhibits intact epidermal layers, minimal necrosis, and a balanced inflammatory response with robust granulation tissue and collagen remodeling, reflecting the synergistic effects of the treatment. In the Lyc-Se-NP (50 mg) group (**F1**,**F2**), the skin structure is well organized with complete preservation of epidermal layers and advanced wound closure. Dense collagen bundles and mature vascular networks are prominent, indicating the optimal therapeutic efficacy of the high-dose combination. Scab (S), granulation tissue (g), dermis (d), epidermis (p), inflammatory cells (m), and arrow mark for neovascularization (red arrow), fibroblast infiltration (black arrow), and fibrous tissue (green arrow)).

The skin section in the untreated group demonstrated delayed healing with persistent fibrin deposition, no granulation tissue, ulceration, scab formation, tissue debris, and inflammatory cell infiltration. The epidermis exhibited necrotic areas and a loss of epithelial integrity (Figure 9(A1)). The dermis had a severe inflammatory infiltrate featuring neutrophils and macrophages (Figure 9(A2)).

In contrast, in the gentamicin group, there was evidence of a more advanced healing process at the wound site, including the initial formation of granulation tissue and early collagen deposition, indicating improved wound healing. The epidermis showed moderate re-epithelialization, with a few layers of epithelial epidermal cells formed under the scab (Figure 9(B1)). The dermis exhibited an insignificant inflammatory response with fewer neutrophils (Figure 9(B2)).

In the lycopene-only group, the epidermis exhibited mild preservation of its architecture, with fewer necrotic areas. The dermis showed a mild to moderate inflammatory response, with a reduced presence of neutrophils and macrophages (Figure 9(C1)). The wound site showed enhanced granulation tissue formation and improved collagen organization, indicating accelerated wound closure (Figure 9(C2)).

Similarly to the lycopene-only group, in the free Se-NP group, the epidermis displayed preserved layers, with one to two layers of degenerated epithelial epidermal cells formed under the scab. The dermis showed a mild inflammatory infiltrate, predominantly lymphocytic (Figure 9(D1)). The wound site exhibited enhanced vascularization and increased collagen synthesis, promoting early tissue repair (Figure 9(D2)).

With Lyc-Se-NP (25 mg) treatment, the epidermis maintained an intact barrier with minimal signs of necrosis and more healthy epidermal layers (Figure 9(E1)). The dermis had a mild inflammatory response, with a balanced presence of neutrophils and macrophages (Figure 9(E2)). The wound site exhibited robust granulation tissue formation and collagen remodeling, indicating the synergistic effects of Lyc and selenium.

Moreover, when treated with Lyc-Se-NPs (50 mg), the skin had a well-organized architecture, with the three main layers (epidermis, dermis, and hypodermis) distinguishable. Plenty of sebaceous glands and hair follicles were in various stages of maturation. The epidermis showed complete preservation of its layers, with no signs of necrosis. The normal keratinization process over the entire wound confirmed the full differentiation of keratinocytes (Figure 9(F1)). The wound site showed advanced closure, with dense collagen bundles and mature vascular networks (Figure 9(F2)), indicating the optimal therapeutic efficacy of Lyc-Se-NPs (50 mg). The semiquantitative scoring of wound healing parameters is presented in Table 6.

#### 2.7.5. Masson’s Trichrome Examination for Dermal Collagen (%) in Skin Sections

**Figure 10:**
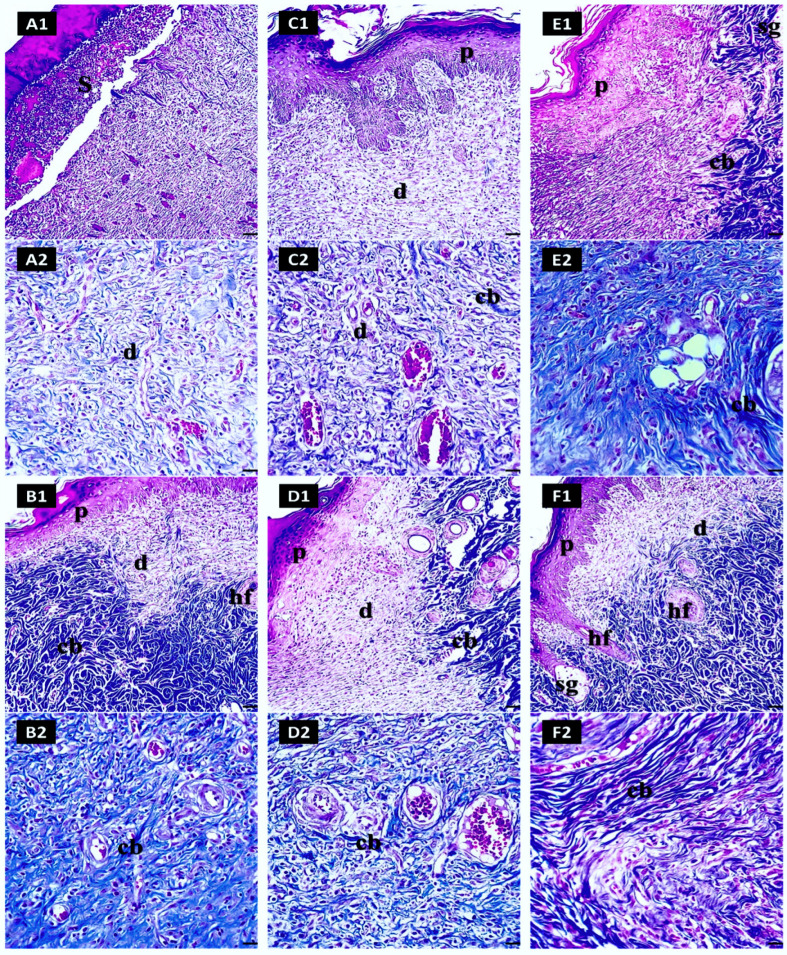
Histopathological assessment of wound healing across different treatments. The untreated group (**A1**,**A2**) exhibits a disrupted epidermal structure, poor collagen deposition, and disorganized fibers, reflecting inadequate wound repair. The gentamicin (**B1**,**B2**) group shows partial preservation of the epidermis, initial collagen deposition, and improved granulation tissue organization, indicating better healing. The lycopene-only group (**C1**,**C2**) has enhanced epidermal preservation and improved collagen organization, suggesting accelerated wound repair. The free Se-NP group (**D1**,**D2**) demonstrates an intact epidermis, minimal necrosis, and improved collagen synthesis. The Lyc-Se-NP (25 mg) group (**E1**,**E2**) shows complete epidermal preservation, robust granulation, and dense collagen bundles, indicating a synergistic effect of the treatments. The Lyc-Se-NP (50 mg) group (**F1**,**F2**) exhibits optimal healing with a well-preserved epidermis, advanced collagen deposition, and mature vascular networks, underscoring the efficacy of the high-dose combination. (p) epidermis, (d) dermis, (hf) hair follicles, (sg) sebaceous gland, (cb) collagen bundles, (S) scab.

In the untreated group, the skin’s outer layer (epidermis) showed a disrupted structure with a significant loss of epithelial integrity (Figure 10(A1)). The layer underneath (dermis) displayed insufficient collagen deposition and was mostly made up of fibrin with a lack of organized granulation tissue. The collagen fibers were poorly oriented and disorganized (Figure 10(A2)). In contrast, the gentamycin group showed partial preservation of epidermal architecture, with early re-epithelialization evident. Wound sites displayed the onset of collagen deposition (Figure 10(B1)). They improved connective tissue organization, with more orderly granulation tissue beginning to form, indicative of enhanced wound healing compared to the negative control (Figure 10(B2)).

The epidermis in the lycopene-only group exhibited better preservation with minimal necrosis (Figure 10(C1)). Enhanced collagen organization and granulation tissue formation at the wound site suggested accelerated wound closure and tissue repair (Figure 10(C2)). Similarly, the free Se-NP group displayed an intact epidermal barrier (Figure 10(D1)), with minimal necrosis and improved collagen synthesis and maturation, contributing to early tissue repair and wound closure (Figure 10(D2)).

**Figure 11:**
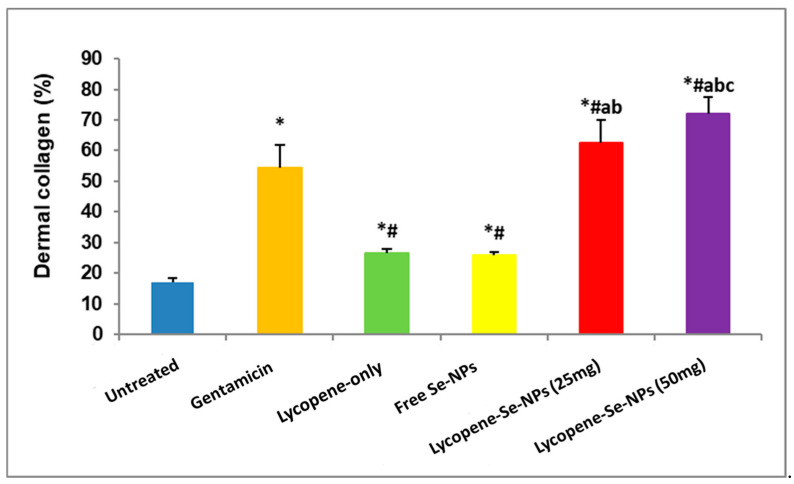
Dermal collagen (%) of skin sections. Data were recorded as mean ± SD (*n* = 6). * Significant vs. untreated group, ^#^ significant vs. gentamicin group, ^a^ significant vs., lycopene-only group, ^b^ significant vs. free Se-NP group, and ^c^ Lyc-Se-NPs (25 mg). Se-NPs: selenium nanoparticles. Each group differed significantly from the others at *p* < 0.05.

The Lyc-Se-NP (25 mg) group showed a fully preserved epidermis with no necrotic areas (Figure 10(E1)), robust granulation tissue formation, and dense collagen bundles (Figure 10(E2)). This indicates a synergistic effect of lycopene and selenium in promoting wound healing. Similarly, the Lyc-Se-NP (50 mg) group maintained an intact epidermis with no necrotic areas (Figure 10(F1)), and the dermis showed advanced closure with dense collagen deposition and well-developed vascular networks (Figure 10(F2)), highlighting the optimal therapeutic effectiveness of Lyc-Se-NPs (50 mg). The results of our dermal collagen (%) evaluation are presented in Figure 11.

#### 2.7.6. Immunohistochemical Staining of NF-κB-p65 in Skin Sections

**Figure 12:**
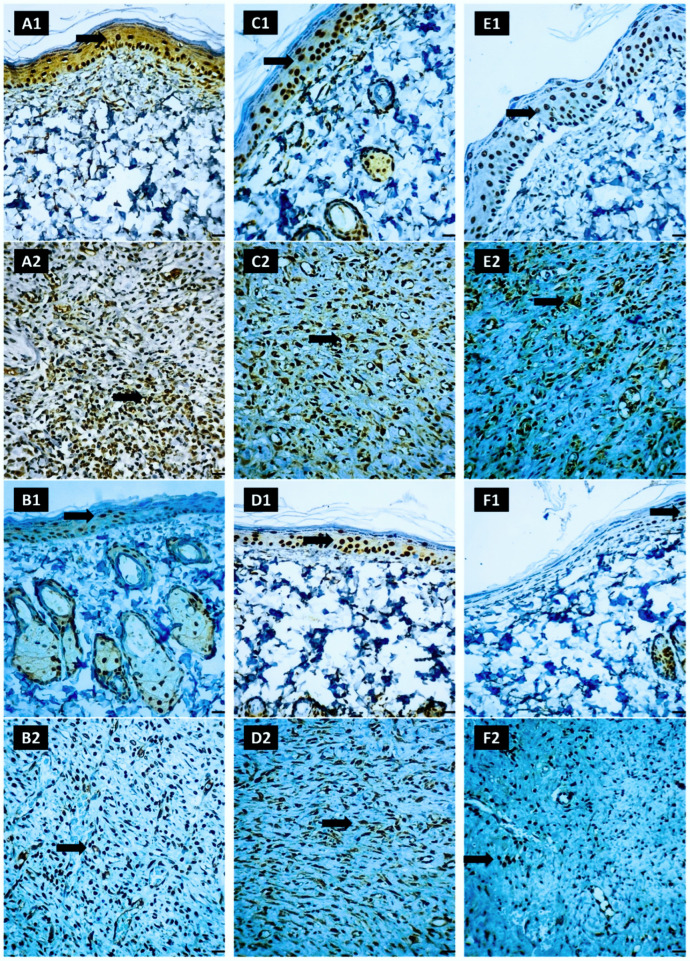
Analysis of NF-κB-p65 activation across different treatment groups. (**A1**,**A2**) show the untreated group with marked NF-κB-p65 activation, characterized by the highest staining and expression scores, indicating robust NF-κB activity. (**B1**,**B2**) depict the gentamicin group with mild NF-κB-p65 activation, evidenced by lower staining and expression levels, reflecting a partial response. (**C1**,**C2**) illustrate the effect of lycopene only, while (**D1**,**D2**) showed the effect of free Se-NPs, both resulting in moderate NF-κB-p65 activation with reduced staining and expression scores. (**E1**,**E2**) present the Lyc-Se-NP (25 mg) group, which further reduced NF-κB-p65 activation but not as effectively as the Lyc-Se-NP (50 mg) group. Finally, (**F1**,**F2**) demonstrate the Lyc-Se-NP (50 mg) group, with the lowest NF-κB-p65 activation, characterized by minimal staining and expression levels, indicating effective suppression of NF-κB-p65 activity. Black arrows indicate positive immunohistochemical expression.

Distinct variations in NF-κB-p65 activation across different treatment groups were observed when analyzing NF-κB-p65 staining as well as expression in the epidermis. The untreated group (Figure 12(A1,A2)) demonstrated a marked activation of NF-κB-p65, characterized by the highest staining and expression scores, indicating robust NF-κB-p65 activity. The gentamicin group (Figure 12(B1,B2)) showed mild NF-κB-p65 activation, with minor staining and expression levels reflecting a partial response. In contrast, treatments with lycopene only (Figure 12(C1,C2)) or free Se-NPs (Figure 12(D1,D2)) resulted in moderate NF-κB-p65 activation, as evidenced by lower staining and expression scores. In the Lyc-Se-NP (25 mg) group (Figure 12(E1,E2)), NF-κB-p65 activation was reduced further, but not as significantly as it was with the high-dose treatment. Notably, the Lyc-Se-NP (50 mg) group (Figure 12(F1,F2)) exhibited the lowest NF-κB-p65 activation, with minimal staining and expression levels, suggesting an effective suppression of NF-κB-p65 activity. The average scores and classification of NF-κB-p65 are presented in Table 7.

## 3. Discussion

One of the several tetraterpene compounds included in tomatoes and tomato-based products is lycopene, a member of the carotenoids. It is widely acknowledged to be a strong antioxidant and a carotenoid that is not pro-vitamin A [22]. An investigation of a population in the United States found that lycopene is the predominant carotenoid in plasma and tissues [23]. Lycopene is beneficial in the management of inflammatory events, oxidative-stress-associated illness, diabetes mellitus, cardiovascular problems, and hepatic, neurological, reproductive, and skeletal disorders [23]. However, its application as a therapeutic agent has been severely limited due to its sensitivity to oxidizing agents, light, and temperature; difficulties dissolving in water; low solubility in oil at ambient temperature; and insufficient bioavailability [23].

The results demonstrated relatively small nanoparticles with an average hydrodynamic diameter of 126.6 ± 3.12 nm, which was confirmed by TEM, showing a smooth spherical particle (90 nm in diameter). Furthermore, the nanoparticles were adequately monodispersed, as indicated by the PDI value (0.3271 ± 0.023), and they had acceptable stabilities, as illustrated by the zeta potential (−23.47 ± 7.11 mV). Furthermore, FTIR illustrated the interaction between poloxamer 407 and lycopene with the appearance of a peak at 2924.22 cm^−1^, which could be explained by carbon–hydrogen SP3 bending. As such, FTIR provided great evidence of the formation of metal nanoparticles, as illustrated by the decreases in the percentages of transmittance of almost all functional groups [24].

The results of this study showed that the Lyc-Se-NPs antioxidant, antibacterial, and anti-inflammatory efficacy exceeded those of free Se-NPs and lycopene.

Selenium supplements offer several health advantages, including protection against cancer, heart disease, diabetes, inflammation-related disorders, and the antioxidant system [25,26]. Regarding different selenium forms, reducing selenium to the nanoparticle form may lessen its toxicity. According to a prior study using a mouse model, selenium nanoforms are less harmful than their inorganic and organic counterparts, as demonstrated by the findings that animals treated with Se-NPs experienced less hepatic damage, fewer bone marrow cell deaths, and less DNA damage [27,28]. Moreover, it has been found that the selenium nanoform exhibits a greater antioxidant capacity than the elemental form.

Meanwhile, the most powerful antioxidant is lycopene. It is a crucial reactive oxygen species (ROS) deactivator. As an example, it can eliminate singlet oxygen two and ten times more efficiently than alpha-tocopherol and beta-carotene, respectively [29]. The health advantages of lycopene have garnered significant attention in recent times. Not only is it a strong antioxidant, but several published reviews and meta-analyses have also assessed and authorized its therapeutic efficacy in preventing and treating a broad range of disorders [30]. Moreover, previous research demonstrated that the presence of active ingredients like lycopene is responsible for tomato extract’s antibacterial action against a variety of bacteria [27].

Concerning the antibacterial activity of the tested compounds, Lyc and Lyc-Se-NPs showed potential activity against *S. aureus*, with MIC values ranging from 256 to 1024 and 64 to 1024 µg/mL, respectively. Moreover, *S. aureus* treated with sub-MICs of Lyc and Lyc-Se-NPs showed significant antibiofilm action.

It is widely recognized that there are three main stages to the healing of a wound following hemostasis. In the initial stage, inflammation takes place, leading to the accumulation of neutrophils and macrophages at the site of the lesion. The proliferation stage comes next, during which re-epithelization, angiogenesis, granulation tissue development, and fibroplasia occur [28]. Ultimately, the remodeling phase is characterized by neovascularization and the onset of neocollagenesis. In this last stage, the wound heals more slowly. Reducing the healing time and avoiding negative side effects like scarring are the two main objectives of wound-healing interventions [28].

According to reports, sepsis brought on by a secondary bacterial infection, possibly by *P. aeruginosa* or *S. aureus*, can make wound healing extremely difficult. Furthermore, through the activation of inflammatory cytokines, inflammation plays a critical role in wound healing [31]. Pro-inflammatory cytokines have a crucial role in initiating inflammation, drawing out neutrophils, eliminating contaminants and pathogens from the site of the injury, and promoting the formation of metalloproteinases (MMPs). These MMPs are involved in the healing process by destroying damaged extracellular matrices (ECMs) to facilitate tissue restoration [32].

Conversely, prolonged inflammation may exacerbate tissue devastation, leading to chronic wounds, as the produced proteinases and cytokines may magnify tissue destruction [33]. Furthermore, inflammation severely devastates the surrounding tissues, and the injury may develop into a pathological condition that deserves more intensive care [33]. Therefore, anti-inflammatory medications are of great importance in helping treat wounds.

In addition to scavenging singlet ROS, lycopene also inhibits lipid peroxidation. It was demonstrated that HO-1 mRNA was increased by lycopene at 50 and 100 μM, but lycopene was unable to control the expression of COX-2 or NOS2 mRNA. Nevertheless, these doses inhibited the production of the LPS-stimulated COX-2, NOS2, and TNF-α genes in RAW264.7 cells [34]. Furthermore, lycopene protects against inflammation caused by β-amyloid. β-amyloid boosted the expression levels of TLR4, NF-κB p65 mRNA, and IL-6β, and the serum levels of IL-1β, TNF-α, and protein at the choroid plexus. Supplementation with lycopene reduced the levels of inflammatory cytokines, reversed the expression of Aβ1-42, and upregulated TLR4, NF-κB p65 mRNA, and protein at the choroid plexus [35].

In carrageenan-induced inflammation, lycopene supplementation (12.5 mg/kg BW) significantly reduced edema in Swiss mice, as determined by immunostaining for NF-κB, iNOS, and COX-2, and using different phlogistic chemicals. Additionally, it reduced the MPO concentration and leukocyte motility in paw tissue and the peritoneal cavity while raising GSH levels [34].

Additionally, studies have investigated lycopene at various levels (i.e., 0.5, 1.0, 2.0, 4.0, 8.0, 10.0, and 25 μM) for its potential to prevent inflammation brought on by cigarette smoking. Increases in TNF-α, interleukin-10, and interferon-γ concentrations were all reduced by lycopene [36]. In a recent study, Sprague Dawley rats were given a single 200 μg injection of LPS to elicit endotoxin-induced uveitis. Treatment with 10 mg/kg of lycopene intraperitoneally significantly reduced the raised total protein concentration, infiltrating cell number, NO, TNF-α, and IL-6 caused by LPS [37].

## 4. Materials and Methods

### 4.1. Drugs and Chemicals

Lycopene capsules (40 mg) were obtained from Puritan’s Pride Inc., Ronkonkoma, NY, USA. Poloxamer 407 USP/NF was sourced from BASF Corporation, Florham Park, NJ, USA. We obtained sodium selenite from Lanxess, Netherlands. L-ascorbic acid was sourced from Research-Lab FINE Chem Industries, Mumbai, India. n-hexane and acetone were obtained from El-Nasr Pharmaceutical Chemicals Co., Cairo, Egypt. Gentamicin was sourced from Sigma-Aldrich, Saint Louis, MO, USA. The highest analytical-grade chemicals were used in this study.

### 4.2. In Vitro Study

#### 4.2.1. Preparation of Selenium Nanoparticles

Lyc-Se-NPs were synthesized by the chemical reduction method utilizing ascorbic acid as a reducing agent and poloxamer 407 as a stabilizer. In brief, the equivalent weight of 40 mg lycopene was dissolved in 3 mL acetone.

Poloxamer 407 (40 mg) was dissolved in 10 mL cold water (4 °C) and was added to 5 mL of sodium selenite (8 mg/mL) mixed with a magnetic stirrer (Stuart, Caliber Scientific, Holland, OH, USA) at 200 rpm for 5 min. Lycopene solution was gradually dropped manually with the aid of a 3 mL plastic syringe, with continuous stirring for an additional 10 min. The solution was then incubated in an ice bath and allowed to equilibrate for 10 min (4 °C). Ascorbic acid (80 mg) was dissolved in 5 mL of distilled water. Using a 5 mL plastic syringe, ascorbic acid was introduced stepwise with continuous stirring until the appearance of a reddish color. The solution was allowed to equilibrate for 2 h, and then the solution was subjected to a probe sonicator (Sonic Vibra Cell, Newtown, CT, USA) for a further 5 min (10 s pulse and 3 s pause) at 69% of its power (130 W), (the solution was incubated in an ice bath).

The colloid dispersion was lyophilized for further investigation. The drug-free nanoparticles were formulated by this same method, except that the addition of lycopene solution was replaced with 3 mL of acetone.

#### 4.2.2. Drug Content

The weight equivalent to 10 mg lycopene was transferred to a 10 mL volumetric flask complete to volume with n-hexane. The UV spectrophotometric scanning was performed utilizing n-hexane as a blank. Lycopene has three peaks of absorbance: 444, 471, and 503 nm [38]. The absorbance of lycopene was investigated at 471 nm, and serial dilution was performed to prepare a standard curve (0.3 to 1.6 µg/mL). The drug content was studied by determining the lycopene absorbance utilizing the UV-VIS spectrophotometric method at 471 nm.

#### 4.2.3. Characterization of Selenium Nanoparticles

##### Average Particle Size, Zeta Potential Evaluation, Polydispersity Index (PDI), SEM and TEM

Particle size and zeta potential are crucial for nanoformulation efficiency, with PDI indicating distribution uniformity [39]. Furthermore, nanoparticles’ cellular uptake, which influences cell and tissue binding, is controlled by their form, dimensions, and zeta potential. Higher zeta potentials result in stronger membrane bonds and increased cellular absorption [40]. The Zeta Sizer Nano (Malvern Panalytical Ltd., Malvern, UK) was used to assess the particle size and zeta potential.

The surface properties and structure of lycopene, drug-free selenium nanoparticles, and Lyc-Se-NPs were verified using the SEM method [41]. One drop of alcoholic solution was then applied to a glass slide. The sample was coated with gold for one minute, and then a 10 kV electron acceleration voltage field emission scanning electron microscope (JEOL, JSM-6510LV, Tokyo, Japan) was used. The size and shape of the Lyc-Se-NPs were evaluated using a transmission electron microscopy (TEM, JEM-2100F, electron microscope, JEOL Ltd., Tokyo, Japan) [41].

##### Fourier Transform Infrared Spectroscopy Analysis (FTIR)

The system’s stability was studied through FTIR analysis, which was carried out to evaluate the interactions or compatibility of the formulations. IR spectra in the range of 4000–400 cm^−1^ were acquired at room temperature using a Perkin–Elmer Fourier transform spectrometer [42] (Bruker, Billerica, MA, USA), and we used FTIR spectra of the lycopene powder, Selenium nanoparticles, and the lycopene-Se-NPs were examined.

### 4.3. Antioxidant Activity Assay

#### 4.3.1. DPPH Radical Scavenging Assay

The DPPH assay was employed for lycopene, free Se-NPs, and Lyc-Se-NPs at different concentrations (1, 2, 4, 6, 8, 10, 12, 14, 16, and 18 µg/mL) using the method described by [43]. A spectrophotometer (Biosystem 310 plus) was employed to measure the absorbance at 517 nm. The reference chemical was ascorbic acid. By utilizing the log dosage inhibition curve (*n* = 3), the IC50 value was determined.

The percentage of DPPH scavenging effect was determined as follows:

$$\%\;\mathrm{Inhibition}=\frac{A0-A1}{A0}\times 100$$

where A_0_ denotes the absorbance of the control response, and A_1_ represents the absorbance in the presence of a test or standard sample.

#### 4.3.2. ABTS Radical Scavenging Assay

ABTS radical scavenging activity was determined for lycopene, free Se-NPs, and Lyc-Se-NPs at different concentrations (1, 2, 4, 6, 8, 10, 12, 14, 16, and 18 µg/mL), following [43] with minor modifications. After incubating for six minutes, the absorbance at 734 nm was measured using a spectrophotometer. The antioxidant activity was assessed as follows:

$$\%\;\mathrm{Inhibition}=\frac{A\;control-A\;sample}{Acontrol}\times 100$$

where A control represents the absorption of the negative control with solution preparation, and A sample represents the sample absorbance after 6 min.

The chemical utilized as the reference was gallic acid. The IC_50_ value was determined using a graph that displayed the sample concentration needed to scavenge 50% of the ABTS free radicals (*n* = 3).

### 4.4. Screening the In Vitro Antibacterial Activity of Lycopene (Lyc) and Lycopene Selenium Nanoparticles (Lyc-Se-NPs)

#### 4.4.1. Microorganisms

The bacterial reference strains were *Escherichia coli* (ATCC 25922), *Staphylococcus aureus* (ATCC 25923), *Proteus mirabilis* (ATCC 35659), *Klebsiella pneumoniae* (ATCC 700603), and *Pseudomonas aeruginosa* (ATCC 27853). A total of 20 *Staphylococcus aureus* clinical isolates were obtained from the culture collection of the Microbiology and Immunology Department, Faculty of Pharmacy, Tanta University.

#### 4.4.2. Agar-Well Diffusion Technique

In the present study, bacterial reference strains were employed to demonstrate the antibacterial activity of lycopene (Lyc), selenium, and lycopene selenium nanoparticles (Lyc-Se-NPs). The screening of the antibacterial activity of Lyc, selenium, and Lyc-Se-NPs proceeded with us establishing cups in Muller–Hinton agar using the agar-well diffusion technique. Bacterial suspensions were prepared to 0.5 McFarland standard in 0.85% saline. Each bacterial reference strain was streaked on a plate with five cups to test the effects of Lyc, selenium, and Lyc-Se-NPs at concentrations of 1000 µg/mL, whereas the other two cups contained gentamicin and DMSO [44].

Antibiotic susceptibility determinations for bacterial isolates were performed according to the Clinical Laboratory Standards Institute (CLSI, 2015 [45]) with the quality control reference strain *Staphylococcus aureus* ATCC 25923. All experiments were conducted in triplicate, and the data are presented as the mean ± standard deviation (SD).

#### 4.4.3. Broth Microdilution Assay

The minimum inhibitory concentration (MIC) values (µg/mL) of Lyc, selenium, and Lyc-Se-NPs against the tested bacterial isolates were determined using the broth microdilution method in a 96-well microtitration plate [46]. The quality control reference strain *Staphylococcus aureus* ATCC 29213 was used. All experiments were conducted in triplicate.

#### 4.4.4. Growth Curves of *S. aureus* Clinical Isolates (Before and After the Treatment with Tested Compounds)

*Staphylococcus aureus* clinical isolates (*n* = 20) were cultivated in LB broth at 37 °C with and without ½, ¼, and ⅛ MICs of Lyc, selenium, and Lyc-Se-NPs. The OD value for treated and untreated isolates was kept at 0.3, and the absorbance of samples collected every 30 min from each culture was measured at 600 nm [41].

### 4.5. In Vitro Antibiofilm Activity

#### 4.5.1. Crystal Violet (CV) Microtiter Plate Assay

The effects of either Lyc, selenium, or Lyc-Se-NPs on *S. aureus* clinical isolates’ biofilm were investigated, as described by [41]. In summary, the isolates were cultivated in TSB containing 1% glucose for 24 h at 37 °C with and without sub-MICs (½, ¼, and ⅛ MICs) of either Lyc, selenium, or Lyc-Se-NPs using a 96-well microtiter plate. The formed biofilm was fixed with absolute methanol for 20 min, followed by drying. After that, the staining of biofilm was performed using 200 µL of 0.1% CV for 15 min and then rinsing with water, and the plate was left to dry. The stained biofilms were dissolved with 200 µL glacial acetic acid (33% (*v*/*v*)). Each experiment was executed in triplicate.

The absorbance was evaluated using a microplate reader (Sunrise TM, TECAN, Switzerland) at 595 nm to determine the optical density (OD). If OD ≤ ODc, the isolate was recorded as a non-producer; ODc < OD ≤ 2 × ODc as a weak producer; 2 × ODc < OD ≤ 4 × ODc as a moderate producer; and 4 × ODc < OD as a strong producer [41]. The percentage reduction in biofilm formation was determined as follows:

$$Percentage\;reduction\;in\;biofilm\;formation=\frac{Control\;OD595\;nm-Treated\;OD595\;nm}{Control\;OD595\;nm}\times \;100$$

#### 4.5.2. Testing Biofilm Thickness and Bacterial Viability Using CLSM

The effects of Lyc, selenium, or Lyc-Se-NPs on the formation of bacterial biofilms were identified using confocal laser scanning microscopy (CLSM). Glass slides were submerged in the *S. aureus* inoculated TSB media without and with ¼ MICs of Lyc, Se, or Lyc-Se-NPs in 6-well plates with a flat bottom. The microplates were incubated at 37 °C for 18 hrs. Afterward, they were treated twice with PBS and stained with 5 µL of acridine orange (AO) and propidium iodide (PI). When living cells were stained, green fluorescence was created, which was visible for 15 min in the absence of light. Red fluorescence was produced when staining dead cells. Finally, we investigated the thickness of the biofilm with CLSM (DMi8, Leica Microsystem, Deerfield, USA) [41].

### 4.6. In Vivo Study

#### 4.6.1. Animals

A national research center in Egypt provided adult male Wister albino rats weighing 200–220 g. The animals were given regular food and unlimited access to water. This study complied with ethical standards for animal care as well as institutional norms. The University of Tanta’s Faculty of Pharmacy research ethics committee, which followed the ARRIVE 2.0 criteria (Code of Protocol: TP/RE/6/23 p-0070), accepted the study protocol.

#### 4.6.2. Wound Model and Experimental Groups

A total of thirty-six rats were randomly assigned to six different groups consisting of six rats each:

(1) Untreated group (infected wound only);
(2) Gentamicin group (infected wound + standard drug gentamycin treatment);
(3) Lycopene-only group (infected wound + lycopene treatment);
(4) Free Se-NP group (infected wound + free Se-NP treatment);
(5) Lyc-Se-NP group (25 mg) (infected wound + Lyc-Se-NP (25 mg) treatment);
(6) Lyc-Se-NP group (50 mg) (infected wound + Lyc-Se-NP (50 mg) treatment).

After isoflurane anesthesia was administered, a small section of each rat’s back was meticulously shaved. The dorsal skin was injured via full-thickness excisional skin cuts according to a technique previously described [47]. Different treatments were applied topically to the surface of the wound for seven days.

#### 4.6.3. Assessment of Wound Closure Rates (WCRs)

Animals were placed on a white plastic platform, and pictures of their wounds were taken. Morphometric software (Version 1.5) was used to examine the photos (Image J^®^, National Institute of Health, Bethesda, MD, USA), to measure the wound areas on days 0, 2, 4, and 7. The WCR was determined as follows:

WCR = (iWA − fWA/iWA) × 100

where WCR is the wound closure rate, iWA is the initial wounded area (day 0), and fWA is the final wounded area (days 2, 4, and 7). This methodology follows the approach detailed by Santos et al. (2021) [48].

#### 4.6.4. Skin Tissue Sample Collection

Rats were given isoflurane anesthesia. Skin wound tissue samples were collected from each group for histopathological and immunohistochemical examination. The remaining tissues were stored at −80 °C for further analysis.

#### 4.6.5. Determination of Inflammatory Marker (TNF-α, IL-1β, and COX-2) and Anti-Inflammatory Marker (HO-1) Contents in Skin Tissues

The contents of inflammatory markers TNF-α, IL-1β, and COX-2 and anti-inflammatory marker HO-1 in skin tissues were evaluated using various commercial ELISA kits purchased from CUSABIO. Co., Houston, TX, USA (Cat. No. CSB-E11987r, CSB-E08055r, CSB-E13399r, and CSB-E08267r, respectively). The assay procedures were achieved according to the manufacturer’s instructions.

#### 4.6.6. Histopathological Evaluation and Masson’s Trichrome Examination for Dermal Collagen (%) in Skin Sections

Dissected skin wound tissue samples were fixed in 10% neutral buffered formalin. The specimens were sectioned to a thickness of 4 µm after undergoing tissue processing using a standard paraffin embedding technique. The samples were then stained with hematoxylin and eosin (H&E) for light microscopy observation.

Histopathological assessments were conducted using a semiquantitative scoring system to evaluate various aspects of tissue repair, including necrosis, inflammation, fibrin deposition, granulation tissue formation, collagen remodeling, vascularization, and epithelialization. The inflammation scores were based on the presence of neutrophils, monocytes, and plasma cells. The scoring system used ranged from ND (not detectable) to ++++ (high), with intermediate scores indicating + (mild), ++ (moderate), and +++ (moderate to high). This scoring methodology for necrosis, inflammation, fibrin deposition, granulation tissue formation, collagen remodeling, epithelialization, and vascularization was adapted from [49].

The mean area percentage of collagen fiber deposition was assessed through Masson’s trichrome staining, with the collagen content analyzed semiquantitatively using ImageJ software 1.54, as outlined by [50].

#### 4.6.7. Immunohistochemical Staining of NF-κB-p65 of Skin Sections

The procedure for applying the immunohistochemical staining approach was as stated in [51]. Dewaxed sections were soaked in a 0.05 M, pH 6.8 citric acid buffer solution to extract antigens. Following that, protein blocks were applied to the sections and H_2_O_2_ at 0.3%. The samples were then treated with NF-κB-p65 antibody (Santa Cruz, CA, USA, Cat No. sc-166416, 1:100 dilution). After that, half an hour was spent at 37 °C using the secondary antibody coupled with horseradish peroxidase.

The sections were exposed for three minutes to the 3,3′-diaminobenzidine tetrahydrochloride reagent. Finally, Mayer’s hematoxylin was used to counterstain the slides. DPX mounting and a wash with distilled water came next. The slides were examined using an Olympus CX21 microscope (Tokyo, Japan).

The immunohistochemical grading was performed semiquantitatively as described by [52] with the average scores used for analysis. The scoring system was defined as follows: 0 = no staining, 1 = light brown–yellow, 2 = brown, and 3 = dark brown staining. Ten fields were evaluated on each slide at 400x magnification. The average positive expression on each slide was scored as follows: 1 = <25%, 2 = 25 to <50%, 3 = 50 to <75%, and 4 = >75%. The final score for each slide was determined by the product of the positive expression percentage and the degree of staining score, resulting in the following classifications: 0 to 1 point was negative (−), 2 to 3 points were weakly positive (+), 4 to 6 points were moderately positive (++), and >6 points were strongly positive (+++).

### 4.7. Statistical Analysis

The results were shown as the mean ± standard deviation (SD). To compare different groups, a one-way analysis of variance (ANOVA) was employed, and Tukey–Kramer post hoc testing. A significance threshold of *p* < 0.05 was used. GraphPad Prism 5 Software, Inc., San Diego, CA, USA, was employed in order to use to perform the statistical analysis.

## 5. Conclusions

The present study describes, for the first time, the antibacterial and antibiofilm activity of lycopene and lycopene-selenium nanoformulation on *S. aureus*. Our findings demonstrate that Lyc and Lyc-Se-NPs have powerful antioxidant activity which was confirmed with the findings of both DPPH and ABTS radical scavenging experiments. Moreover, they demonstrated significant anti-inflammatory properties, which were evident by the downregulation of TNF-α, IL-1β, and COX-2. Given the unique characteristics of Lyc-Se-NPs, we propose that this formula should be subject to further research in the field of inflammation management.

## Figures and Tables

**Table 1:** Particle size, zeta potential, and polydispersity index (PDI) of lycopene nanoparticles.

Character	Mean ± SD	Range
Particle size (nm)	126.6 ± 3.12	124.6 to 130.2
Zeta potential mV	−23.47 ± 7.11	−16.93 to −31.04
PDI	0.3271 ± 0.023	0.31 to 0.35
Drug content (µg/mL)	31.85 ± 3.33	29.40 to 35.64

**Table 2:** Inhibition zone diameters of the tested compounds against different bacterial strains.

Bacterial Reference Strain	Inhibition Zone Diameter (mm)				
	Lyc	Selenium	Lyc-Se-NPs	Gentamicin	DMSO
*S. aureus* (ATCC 25923)	11.3 ± 0.58 ^#a^	12 ± 0.00 *^#a^	17.3 ± 0.58 *	18 ± 1.00 *	10 ± 0.00
*E. coli* (ATCC 25922)	11 ± 0.00 ^#^	11 ± 1.00 ^#^	12 ± 0.00 *^#^	20.67 ± 0.58 *	10 ± 0.00
*P. aeruginosa* (ATCC 27853)	11 ± 0.00 ^#^	11 ± 0.00 ^#^	12 ± 1.00 *^#^	20 ± 1.00 *	10 ± 0.00
*P. mirabilis* (ATCC 35659)	10 ± 0.00 ^#^	10 ± 0.00 ^#^	10.67 ± 0.58 ^#^	16.67 ± 0.58 *	10 ± 0.00
*K. pneumoniae* (ATCC 700603)	11 ± 1.00 ^#^	10 ± 0.00 ^#^	11 ± 0.00 ^#^	20 ± 2.00 *	10 ± 0.00

Data were recorded as the mean ± SD (*n* = 3). * Significant vs. DMSO, ^#^ significant vs. gentamicin, ^a^ significant vs. Lyc-Se-NPs. Se-NPs: selenium nanoparticles. Each group differed significantly from the others at *p* ≤ 0.05.

**Table 3:** MIC values of Lyc, selenium, and Lyc-Se-NPs against the tested *S. aureus* clinical isolates (*n* = 20).

MIC (µg/mL)	Number of Isolates (%)			
	Lyc	Selenium	Lyc-Se-NPs	Gentamicin
16	0 (0)	0 (0)	0 (0)	3 (15)
32	0 (0)	0 (0)	0 (0)	5 (25)
64	0 (0)	0 (0)	8 (40)	5 (25)
128	0 (0)	0 (0)	4 (20)	4 (20)
256	3 (15)	0 (0)	5 (25)	2 (10)
512	7 (35)	1 (5)	2 (10)	1 (5)
1024	4 (20)	2 (10)	1 (5)	0 (0)
>1024	6 (30)	17 (85)	0 (0)	0 (0)

**Table 4:** Effect of the treatment by the tested compounds at sub-MICs on the biofilm formation.

Categories of Biofilm Production	Number of Isolates (%)									
	Pre- Treatment	Post-Treatment								
		Lyc			Selenium			Lyc-Se-NPs		
		⅛ MIC	¼ MIC	½ MIC	⅛ MIC	¼ MIC	½ MIC	⅛ MIC	¼ MIC	½ MIC
None	1 (5)	2 (10)	2 (10)	4 (20)	1 (5)	1 (5)	2 (10)	2 (10)	4 (20)	9 (45)
Weak	4 (20)	8 (40)	8 (40)	8 (40)	6 (30)	6 (30)	5 (25)	9 (45)	8 (40)	6 (30)
Moderate	10 (50)	6 (30)	6 (30)	5 (25)	8 (40)	8 (40)	8 (40)	6 (30)	6 (30)	4 (20)
Strong	5 (25)	4 (20)	4 (20)	3 (15)	5 (25)	5 (25)	5 (25)	3 (15)	2 (10)	1 (5)
Total producers	19 (95)	18 (90)	18 (90)	16 (80)	19 (95)	19 (95)	18 (90)	18 (90)	16 (80)	11 (55)

**Table 5:** Average wound closure rates (WCRs).

Groups	Day 2 (%)	Day 4 (%)	Day 7 (%)
Untreated	8.22 ± 0.23	12.58 ± 0.48	20.23 ± 0.43
Gentamicin	17.65 ± 0.19 *	34.05 ± 0.33 *	50.10 ± 0.45 *
Lycopene only	13.05 ± 0.22 *^#^	24.58 ± 0.23 *^#^	36.17 ± 0.62 *^#^
Free Se-NPs	12.15 ± 0.18 *^#^	23.15 ± 0.25 *^#^	35.67 ± 0.15 *^#^
Lyc-Se-NPs (25 mg)	21.17 ± 0.16 *^#ab^	40.08 ± 0.4 *^#ab^	60.35 ± 0.48 *^#ab^
Lyc-Se-NPs (50 mg)	25.13 ± 0.23 *^#abc^	45.22 ± 0.35 *^#abc^	70.23 ± 0.52 *^#abc^

Data were recorded as the mean ± SD (*n* = 6). * Significant vs. untreated group, ^#^ significant vs. gentamicin group, ^a^ significant vs. lycopene-only group, ^b^ significant vs. free Se-NP group, and ^c^ Lyc-Se-NPs (25 mg). Se-NPs: selenium nanoparticles. Each group differed significantly from the others at *p* ≤ 0.05.

**Table 6:** Semi-quantitative scoring of wound healing parameters.

Parameter	Untreated	Gentamicin	Lycopene	Free Se-NPs	Lyc-Se-NPs (25 mg)	Lyc-Se-NPs (50 mg)
Necrosis	++++	++	+++	+++	+	ND
Inflammation	++++	+	++	++	+	ND
Granulation tissue	ND	++	++	++	+++	++++
Vascularization	+	+++	++	++	++	++++
Epithelization	ND	+++	+	+	+++	++++

In the scoring system, ND stands for “not detectable”, + indicates a “mild score”, ++ represents a “moderate score”, +++ denotes a “moderate to high score”, and ++++ signifies a “high score”.

**Table 7:** Average scores and classification.

Group	Average Staining Score	Average Expression Score	Average Final Score	Classification
Untreated	2.6 ± 0.3	3.5 ± 0.42	10.5 ± 1.7	++++
Gentamicin	1.6 ± 0.52 *	2.1 ± 0.23 *	4.4 ± 1.6	++
Lycopene only	1.4 ± 0.12 *^#^	2.6 ± 0.21 *^#^	5.0 ± 1.8	+++
Free Se-NPs	1.5 ± 0.06 *^#^	2.5 ± 0.19 *^#^	7.3 ± 1.0	+++
Lyc-Se-NPs (25 mg)	1.3 ± 0.07 *^#^	1.5 ± 0.04 *^#^	3.0 ± 0.9	++
Lyc-Se-NPs (50 mg)	0.6 ± 0.08 *^#abc^	1.4 ± 0.02 *^#abc^	0.6 ± 0.5	+

Data were recorded as mean ± SD (*n* = 6). * Significant vs. untreated group, ^#^ significant vs. gentamicin group, ^a^ significant vs. lycopene-only group, ^b^ significant vs. free Se-NP group, and ^c^ Lyc-Se-NPs (25 mg). Se-NPs: selenium nanoparticles. Each group differed significantly from the others at *p* < 0.05. ++++: severe, +++ moderate, ++ mild, and + minimal.

## Data Availability

All data generated or analyzed during this study are included in this article.

## Supplementary Materials

The following supporting information can be downloaded at: https://www.mdpi.com/article/10.3390/ph17121600/s1, Table S1. MIC values of tested compounds against *S. aureus* clinical isolates and the effect of the treatment by the tested compounds at sub-MICs on biofilm formation (S; strong producer, M; moderate producer, W; weak producer, N; non-producer); Figure S1. Growth curves of *S. aureus* clinical isolates grown in the absence and presence of ½, ¼, and ⅛ MICs of the tested compounds at different time intervals.
