# Supplementary material for: Investigating the Antibacterial, Antioxidant, and Anti-Inflammatory Properties of a Lycopene Selenium Nano-Formulation: An In Vitro and In Vivo Study
Source: Pharmaceuticals (Basel). 2024 Nov 27;17(12):1600. doi: 10.3390/ph17121600 (PMC11679940; doi:10.3390/ph17121600)

pharmaceuticals

Supplementary Materials

Investigating the Antibacterial, Antioxidant, and Anti-Inflammatory Properties of a Lycopene Selenium Nano-Formulation: An In Vitro and In Vivo Study

Reem Binsuwaidan [1], Thanaa A. El-Masry [2], Maysa M. F. El-Nagar [2,*], Enas I. El Zahaby [3], Mohamed M. S. Gaballa [4] and Maisra M. El-Bouseary [5,*]

[1] Department of Pharmaceutical Sciences, College of Pharmacy, Princess Nourah bint Abdulrahman University, P.O. Box 84428, Riyadh 11671, Saudi Arabia; rabinsuwaidan@pnu.edu.sa (R.B.)

[2] Department of Pharmacology and Toxicology, Faculty of Pharmacy, Tanta University, Tanta 31527, Egypt; thanaa.elmasri@pharm.tanta.edu.eg (T.A.E.); maysa_elnagar@outlook.com (M.M.F.E.)

[3] Department of Pharmaceutics, Faculty of Pharmacy, Delta University for Science and Technology, Gamasa 35712, Egypt; enas.elzahabi@deltauniv.edu.eg (E.I.E.)

[4] Department of Pathology, Faculty of Veterinary Medicine, Benha University, Toukh 13736, Egypt; Mohamed.gaballah@fvtm.bu.edu.eg (M.M.S.G.)

[5] Department of Microbiology and Immunology, Faculty of Pharmacy, Tanta University, Tanta 31527, Egypt; Maysra_mohamed@pharm.tanta.edu.eg (M.M.E.)

Correspondence: Maysra_mohamed@pharm.tanta.edu.eg; Maysa_elnagar@outlook.com

Table S1. MIC values of tested compounds against *S. aureus* clinical isolates and the effect of the treatment by tested compounds at sub-MICs on the biofilm formation. (S; strong producer, M; moderate producer, W; weak producer, N; non-producer).

Isolate code	MIC (µg/ml)				Pre-treatment	Post-treatment								
						Lyc			Selenium			Lyc-Se-NPs		
	Lyc	Selenium	Lyc-Se-NPs	Gentamicin		⅛ MIC	¼ MIC	½ MIC	⅛ MIC	¼ MIC	½ MIC	⅛ MIC	¼ MIC	½ MIC
S1	1024	> 1024	64	16	S	S	S	S	S	S	S	M	M	M
S2	1024	> 1024	128	64	M	W	W	W	M	M	M	W	W	N
S3	512	> 1024	256	128	M	M	M	W	M	M	M	W	W	W
S4	512	> 1024	64	16	N	N	N	N	N	N	N	N	N	N
S5	> 1024	> 1024	64	32	W	W	W	W	W	W	W	W	W	N
S6	1024	> 1024	1024	512	M	M	M	W	M	M	M	W	W	W
S7	512	> 1024	256	128	M	W	W	W	M	M	M	W	N	N
S8	512	> 1024	256	256	S	S	S	S	S	S	S	M	M	M
S9	512	> 1024	512	256	M	W	W	M	M	M	M	M	M	N
S10	> 1024	> 1024	64	16	M	W	W	W	W	W	W	W	N	N
S11	256	512	128	64	W	N	N	N	W	W	N	N	N	N
S12	> 1024	1024	512	128	S	S	S	S	S	S	S	S	S	S
S13	> 1024	> 1024	64	32	W	W	W	N	W	W	W	W	W	N
S14	512	> 1024	256	128	M	W	W	W	W	W	W	W	W	W
S15	> 1024	> 1024	64	32	M	M	M	W	M	M	M	M	M	W
S16	256	1024	128	64	S	S	S	M	S	S	S	S	M	M
S17	1024	> 1024	256	64	M	M	M	M	M	M	M	M	W	W
S18	> 1024	> 1024	64	32	M	M	M	M	M	M	M	M	M	W
S19	512	> 1024	128	64	S	M	M	M	S	S	S	S	S	M
S20	256	> 1024	64	32	W	W	W	N	W	W	W	W	W	N

Supplementary Figure S1. Growth curves of *S. aureus* clinical isolates grown in the absence and presence of ½, ¼, and ⅛ MICs of tested compounds at different interval times.

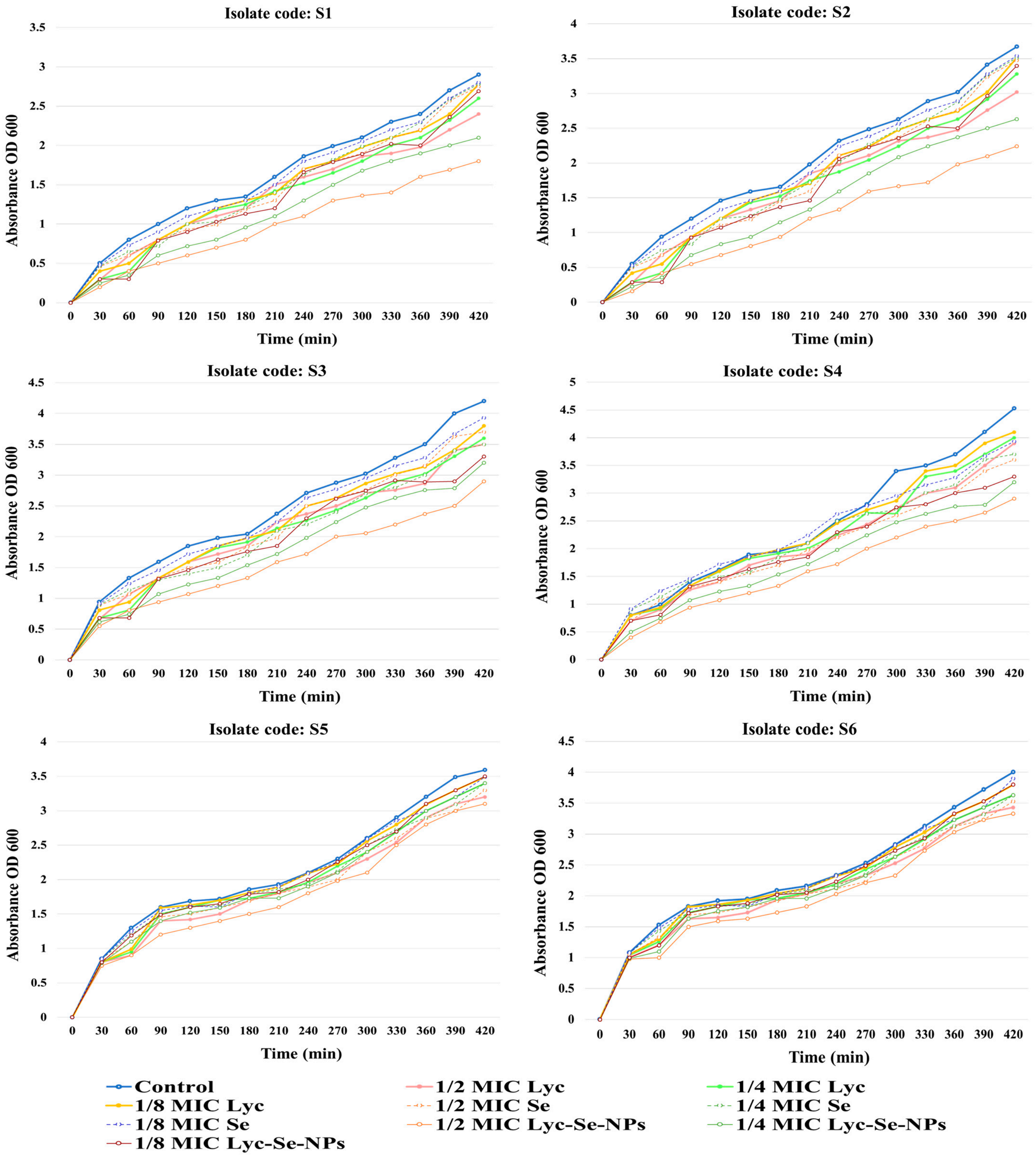

Figure S1 cont.

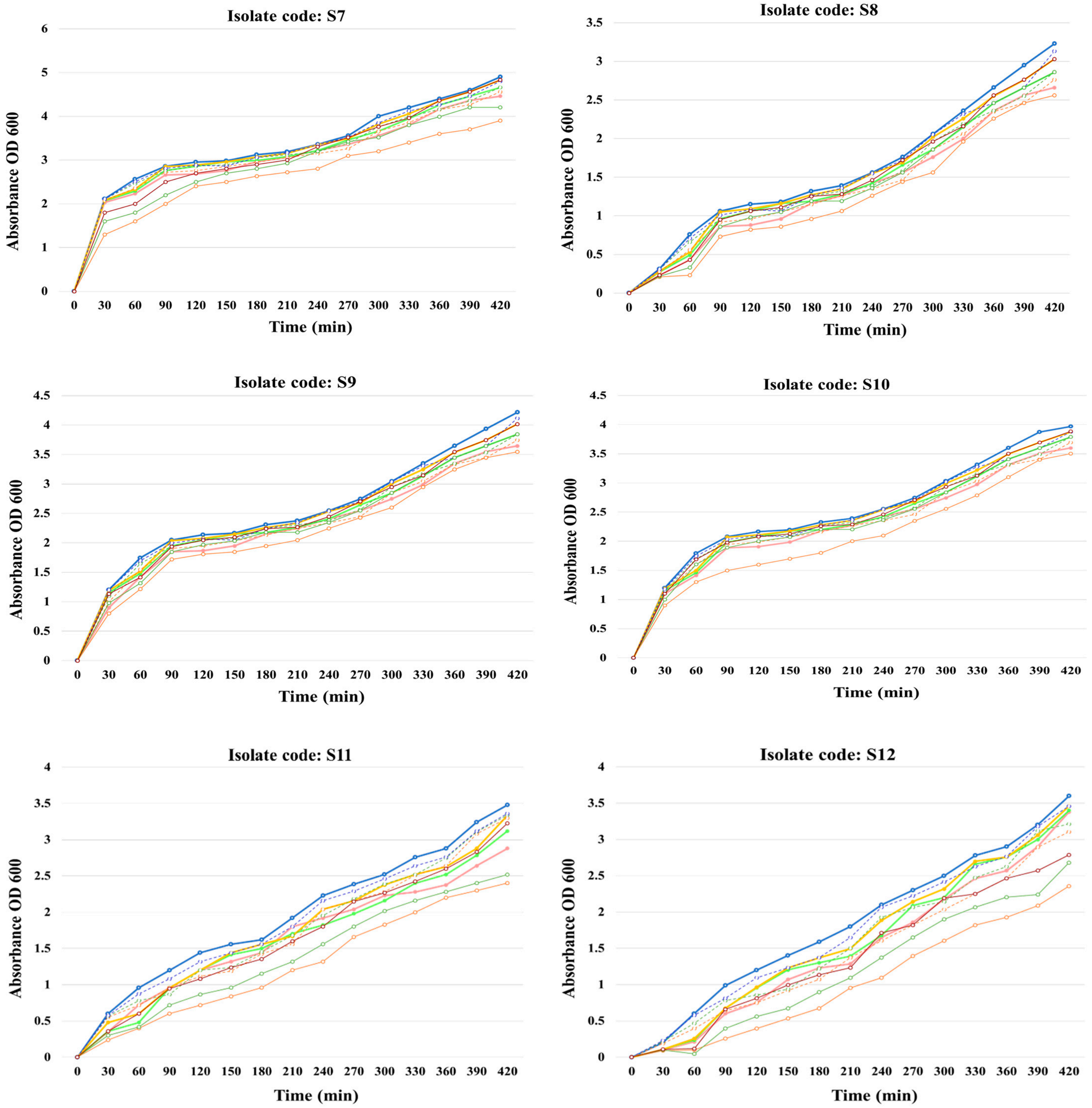

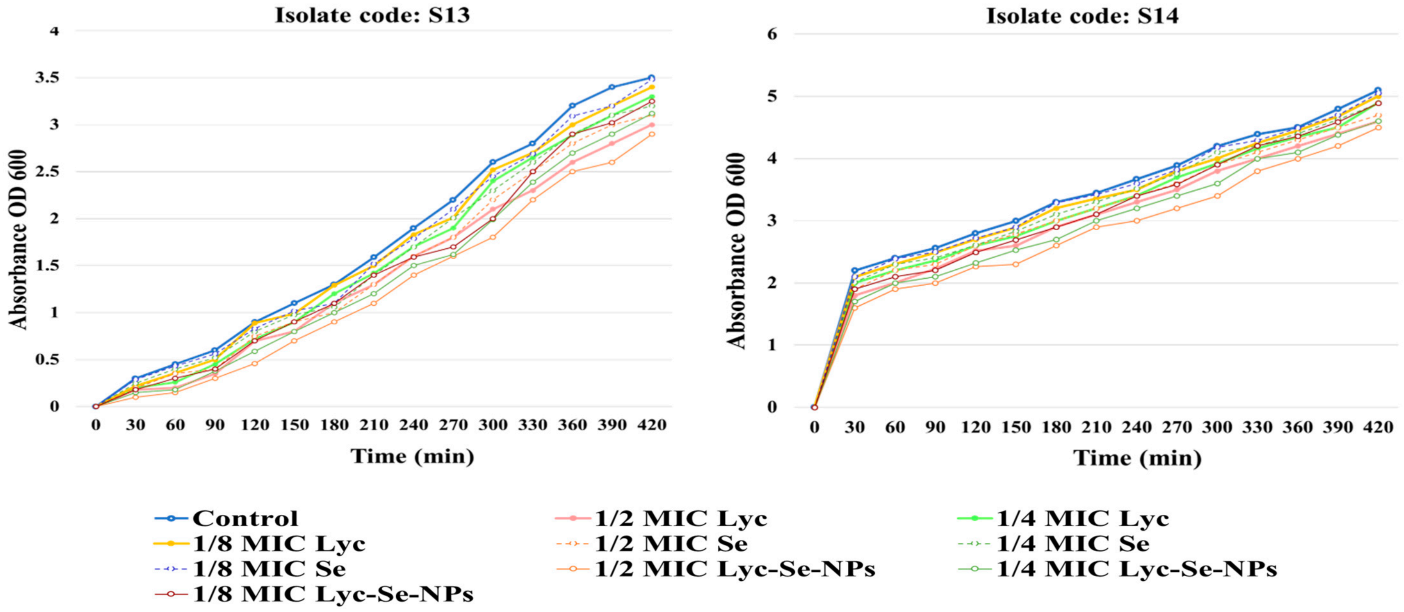
Isolate code: S13
Absorbance OD 600
Time (min)
Isolate code: S14
Absorbance OD 600
Time (min)
Control
1/2 MIC Lyc
1/4 MIC Lyc
1/8 MIC Lyc
1/2 MIC Se
1/4 MIC Se
1/8 MIC Se
1/2 MIC Lyc-Se-NPs
1/4 MIC Lyc-Se-NPs
1/8 MIC Lyc-Se-NPs

Figure S1 cont.

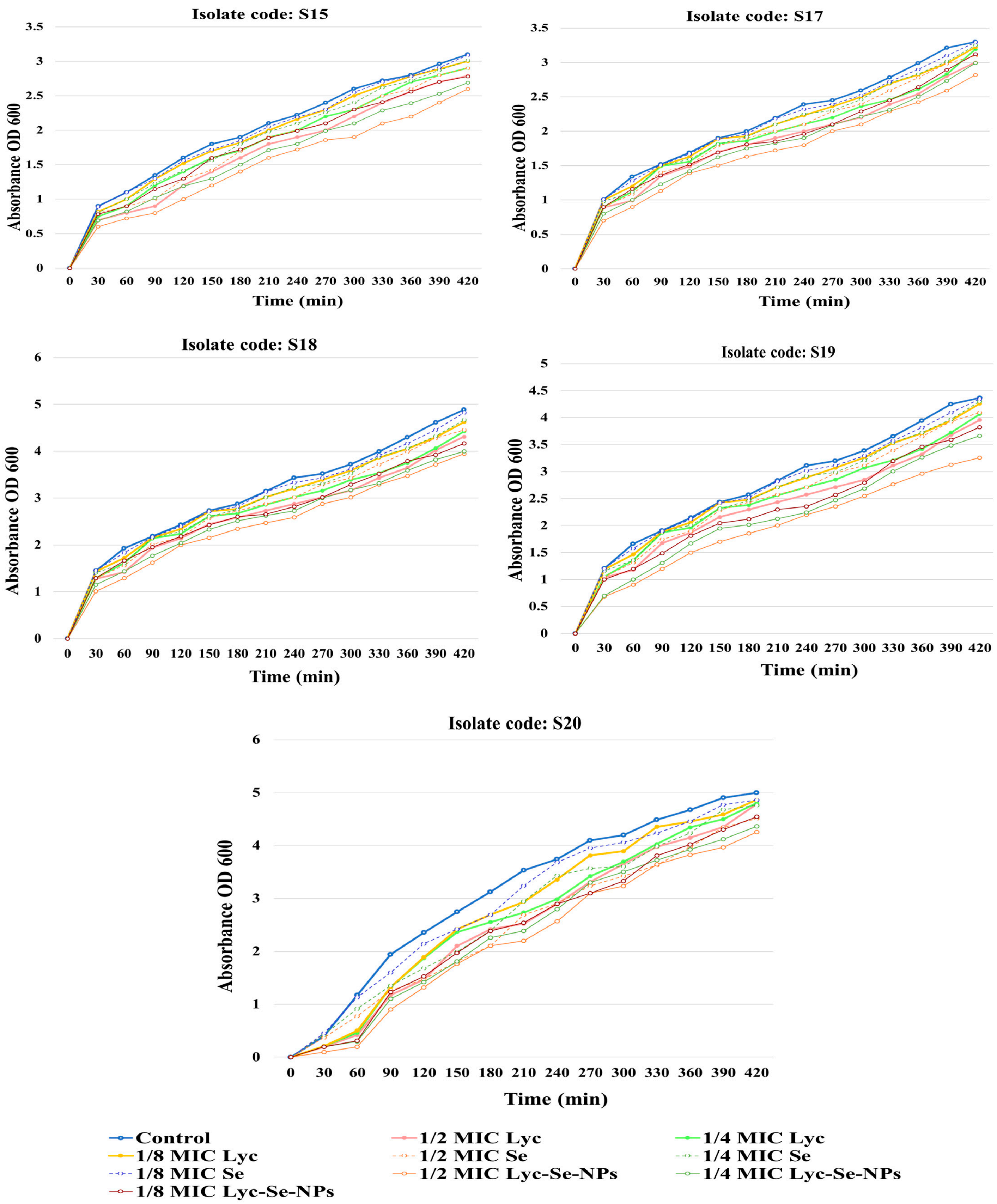

Supplement: Supplementary file 1 [file pharmaceuticals-17-01600-s001.zip › pharmaceuticals-3261349-supplementary.pdf]
